# Polyphenols could be Effective in Exerting a Disinfectant-Like Action on Bioprosthetic Heart Valves, Counteracting Bacterial Adhesiveness

**DOI:** 10.26502/fccm.92920287

**Published:** 2022-09-30

**Authors:** Filippo Naso, Antonio Maria Calafiore, Mario Gaudino, Peter Zilla, Axel Haverich, Andrea Colli, Robert John Melder, Alessandro Gandaglia

**Affiliations:** 1 Biocompatibility Innovation SRL, Este, Padua, Italy; 2 Department of Cardiovascular Sciences, Gemelli Molise, Campobasso, Italy; 3 Department of Cardiothoracic Surgery, Weill Cornell Medicine, New York, USA; 4 Christian Barnard Department of Cardiothoracic Surgery, Groote Schuur Hospital, University of Cape Town, Cape Town, South Africa; 5 Department of Cardiothoracic, Transplantation and Vascular Surgery, Hannover Medical School, Hannover, Germany; 6 Cardiac Surgery Unit, Department of Surgical, Medical and Molecular Pathology and Critical Care, University of Pisa, Pisa, Italy; 7 Mountain Hawk Consulting LLC, Glen Allen, Virginia, USA

**Keywords:** Anti-Microbial Properties, Bioprosthetic Heart Valves, Infective Endocarditis, Polyphenols

## Abstract

**Background:**

The incidence of infective endocarditis in patients with bioprosthetic heart valves is over 100 times that of the general population with *S. aureus* recognized as the causative organism in approximately 1/3 of cases. In this study, (1) the microbicidal and virucidal effect of a polyphenolic solution was carefully evaluated. The same solution was then adopted for the treatment of a commercial bioprosthetic heart valve model for (2) the assessment of inhibition of *S. aureus* adhesiveness.

**Methods:**

(1) the viability of 9 microorganisms strains (colony-forming units) and the infectivity degree of 3 viral strains (cellular infection capacity) were evaluated after suspension in the polyphenolic solution. (2) Leaflets from a treated and untreated commercial surgical valve model were incubated with a known concentration of *S. aureus*. After incubation, the leaflets were homogenized and placed in specific culture media to quantify the bacterial load.

**Results:**

(1) The polyphenolic solution proved to be effective in eliminating microorganisms strains guaranteeing the killing of at least 99.9%. The effectiveness is particularly relevant against *M. chelonae* (99.999%). (2) The polyphenol-based treatment resulted in the inhibition of the S. aureus adhesiveness by 96% concerning untreated samples.

**Conclusions:**

The data suggest an interesting protective effect against infections and bacterial adhesiveness by a polyphenolic-based solution. Further studies will plan to extend the panel of microorganisms for the evaluation of the anti-adhesive effect; however, the use of optimized polyphenolic blends could lead to the development of new treatments capable to make transcatheter-valve substitutes more resistant to infection.

## 1. Introduction

Bacterial infection is one of the causes that contribute to bioprosthetic heart valve (BHV) degeneration, especially in the case of transcatheter BHV. In the industrialized world, the reported incidence of infective endocarditis (IEs) in patients with prosthetic valves is over 100 times that of the general population [1,2] and this risk may be even higher in the elderly group of patients undergoing transcatheter BHV implants who are frequently hospitalized and subjected to invasive procedures coupled with healthcare-associated bacteremia [3]. Prosthetic valve infection has been considered the most severe form of IEs, with *S. aureus* recognized as the causative organism in approximately 1/3 of cases. The overall proportion of IEs due to *S. aureus* in the USA rose from 24% to 32%, supported by the fact that 10% to 20% of the population are persistent carriers of such bacterial strain [4]. *S. aureus*-related endocarditis has been reported as an aggressive disease with increased occurrence of embolism, stroke, persistent bacteremia, and death [5]. Using whole-genome sequencing, Oberbach and colleagues [6] reported that bacterial infiltration was present in 52% of the explanted degenerated heart valves and was localized in calcified structures, indicating a direct involvement in the degenerative process. This last aspect is very important since transcatheter valve-in-valve implantation has shown good results in patients suffering from structural valve deterioration of a previously implanted surgical bioprosthetic heart valve (SBHV). In this case, the dysfunctional valve is not removed, but the transcatheter BHV is deployed inside the compromised valve. This could potentially lead to bacterial migration into and colonization of the new transcatheter bioprosthesis. Comparisons between SBHVs and TBHVs in terms of IE incidence and outcomes are still under investigation [7]. Besides their established antioxidant and anti-inflammatory properties [8,9], many phenolic compounds may exhibit significant antibacterial activity too. In this study, the bactericidal and virucidal effects of a polyphenols-based preparation were evaluated. In addition, the same solution was adopted for the treatment of a commercial BHV model to evaluate a potential bacterial anti-adhesiveness effect.

## 2. Materials and Methods

### 2.1 Polyphenolic Solution Preparation

A blend of polyphenols was solubilized in phosphate buffer solution (PBS, 50mM NaH_2_PO_4_, 20mM Na_2_HPO_4_) at room temperature (RT) as previously described [10,11]. The solution is sterile-filtered with a 0.22 μm filter. Hereafter the polyphenolic solution will be referred to as PS. Commercially pericardial Trifecta-GT BHVs (T-GT, St. Jude Medical/Abbott, Santa Clara, CA, USA) were utilized for the assessment of the protective effects from bacterial adhesiveness comparing PS-treated and untreated isolated leaflets [12]. Before testing, T-GT BHVs were extracted from their packaging and washed in sterile PBS at pH 7.4 and RT for 15 min (three times), as required by the manual for “Preimplantation Directions for Use” provided by the manufacturer. The PS-treated valves were produced using the PS solution according to the treatment that has been previously described [14,16,17]. Briefly, isolated leaflets from T-Gt valves were allowed to drain, rinsed with PBS and transferred to the PS, and left to react under moderate but constant stirring, in the dark, for two-step of 30 minutes each, at RT. At the end of incubation, the tissue is subjected to two washes in isotonic PBS for 15 min each.

### 2.2 Assessment of the Microbicidal Potential of the PS

The microbicidal activity (MA) was evaluated regarding the following different micro-organisms: *Staphyloccoccus aureus* ATCC 6538, *Pseudomonas aeruginosa* ATCC 9027, *Enterococcus faecalis* ATCC 29212, *Listeria monocytogenes* ATCC 19111, *Salmonella enterica typhimurium* ATCC 14028, *Streptococcus viridans* ATCC 6249, a nontuberculous mycobacterium *Mycobacterium chelonae* ATCC 35752, a yeast *Candida albicans* ATCC 10231 and a fungus *Aspergillus brasiliensis* ATCC 16404. MA assay consists of a suspension method with a single incubation for 24 hours of a known concentration bacteria with PS (inoculum). Sterile water, 10% v/v ethanol in PBS, and antibiotic solutions (50μg/ml of Gentamicin for bacterial strains and 1200μg/ml of Nystatin B for mycotic strain) were used as controls. At the end of the incubation time, the content of each test tube is seeded into 90mm sterile Petri dishes in a specific agar medium, with a pour plate or spread plate technique depending on the micro-organisms tested, after dilution in Tryptone Salt Broth (MRD Broth). The plates are incubated at specific conditions and temperatures according to the growth requirements of each microorganism. For each type of microorganism, a microbial suspension in MRD Broth was quantified through the spectrophotometer at 620nm wavelength in a disposable 10mm path length cuvette and the absorbance was measured: the range between 0.150 and 0.460 corresponds to a concentration of cells between 1×10^8CFU/ml and 3×10^8CFU/ml (with *Candida albicans* between 1×10^7CFU/ml and 3×10^7CFU/ml). For *Streptococcus oralis*, as there was no correlation between the absorbance measure and the bacteria concentration, the quantification was performed by cell count at the microscope. Data were elaborated comparing the growth of the microorganisms starting from t0. The percentage of bactericidal activity (Table 1) was determined considering the result obtained by incubating the bacterial inoculum in sterile water at 100% growth.

### 2.3 Assessment of the Virucidal Potential of the PS

The virucidal activity of the PS was assessed according to the guidelines (EN 14476:2013+A2:2019/UNI EN 14476:2019). Quantitative suspension test for the evaluation of virucidal activity in the medical area. Test method and requirements (Phase 2/Step 1). The virucidal assay was performed regarding the following virus strains: Poliovirus Type 1 LSc-2ab (RVB-1260), Adenovirus Type 5 (ATCC VR-5), and Murine norovirus S99 (RVB-651).

### 2.4 Resistance to Tissue Bacterial Adhesion

The anti-adhesive bacterial activity on the PS-treated and untreated T-GT leaflets was evaluated regarding the *Staphylococcus aureus* (*S. aureus*) ATCC 6538 (gram-positive). The bacteria were grown overnight in Tryptic Soy Broth (TSB) at 37°C. The total bacterial load was assessed by 10-factor serial dilutions in TSB (10–1 to 10–7), sown in Petri dishes with appropriate selective medium (MSA-Mannitol Selective Agar), and kept in an overnight incubator. Following incubation, the CFU were counted to determine the effective concentration of the microorganism. Furthermore, the optical density at 600nm was determined from each tiled dilution, to verify the linearity between the latter and the effective microbial load of the broth. PS-treated and untreated T-GT leaflets samples were isolated using a biopsy punch (3mm in diameter, n=5 for each type of sample), to obtain the same effective surface for bacterial adhesion. To eliminate any bacterial load before the adhesiveness test, the punched leaflets samples were washed with PBS and incubated overnight at RT in PBS, supplemented with gentamicin (300μg/mL) under moderate but constant agitation. Following overnight incubation, the leaflet samples were washed extensively in PBS to remove any remaining antibiotics that could skew the test results. Subsequently, the PS-treated (n=6) and untreated (n=6) samples were exposed to *S. aureus* bacterial suspensions (bacterial load 1×10^7^CFU/mL) for 90min at RT under moderate but constant agitation. Subsequently, the samples were subjected to three moderate vortexing passages to facilitate the detachment of the loosely bound bacteria. Finally, the samples were homogenized (Ultra-Turrax, IKA, Germany), and serial dilutions of the obtained homogenates were plated in Petri dishes containing the appropriate selective growth media. Finally, after 24hs of incubation at 37°C, the CFU were counted for each type of sample. The bacterial anti-adhesive activity was calculated using the following formula:

$$100-[(\frac{CT}{CNT})*100]$$

where CT was the bacterial charge of the tissue sample obtained from PS-treated T-GT and CNT was the bacterial charge from untreated ones.

### 2.5 Assessment of the Non-Pyrogenicity of the PS-Treated Tissue

Pyrogenicity was assessed by the monocyte activation test (MAT) as qualified and validated for the detection of pyrogens by the European Center for the Validation of Alternative Methods [13]. Six leaflets from the commercial T-GT BHVs model were isolated and cut in half. For each leaflet, half was subjected to PS-treatment and the remaining part was used as a reference control. Samples were placed for 1 hour in 40ml of endotoxin-free water under moderate shaking at 37°C. The water was analyzed with the MAT test. The resulting cytokine production was then detected using an ELISA immunological assay involving specific antibodies and an enzymatic color reaction. Reference value to be considered pyrogenic: ≤ 20 EU/device [13].

### 2.6 Statistical Analysis

The data was analyzed in Microsoft Excel® and Prism® 7 for Windows (v7.03, GraphPad Software lnc., 157 California) and expressed as mean ± standard deviation (SD). A two-sided unpaired T-test was used to assess significant differences between the treated and untreated groups, at the 0.05 confidence level.

## 3. Results

### 3.1 Assessment of the Microbicidal Potential of the PS

Table 1 reported the percentage of MA of the PS compared to standard antibodies and ethanol solutions. The PS proved to be particularly effective in eliminating various bacterial strains (both Gram+ and Gram-) guaranteeing the killing of at least 99.9% (*A. brasiliensis*). The effectiveness is particularly relevant also against *M. chelonae* (99.999%). PS is much more effective than an ethanol-based solution and comparable to that of standard treatment with antibiotics.

### 3.2 Assessment of the Virucidal Potential of the PS

Table 2 reported the percentage of virucidal activity of the PS diluted to 80% (corresponding to the highest possible concentration feasible according to the method). The treatment resulted in the inactivation of at least 99% of viruses (*Murine norovirus*).

### 3.3 Resistance to Tissue Bacterial Adhesion

**Figure 1:**
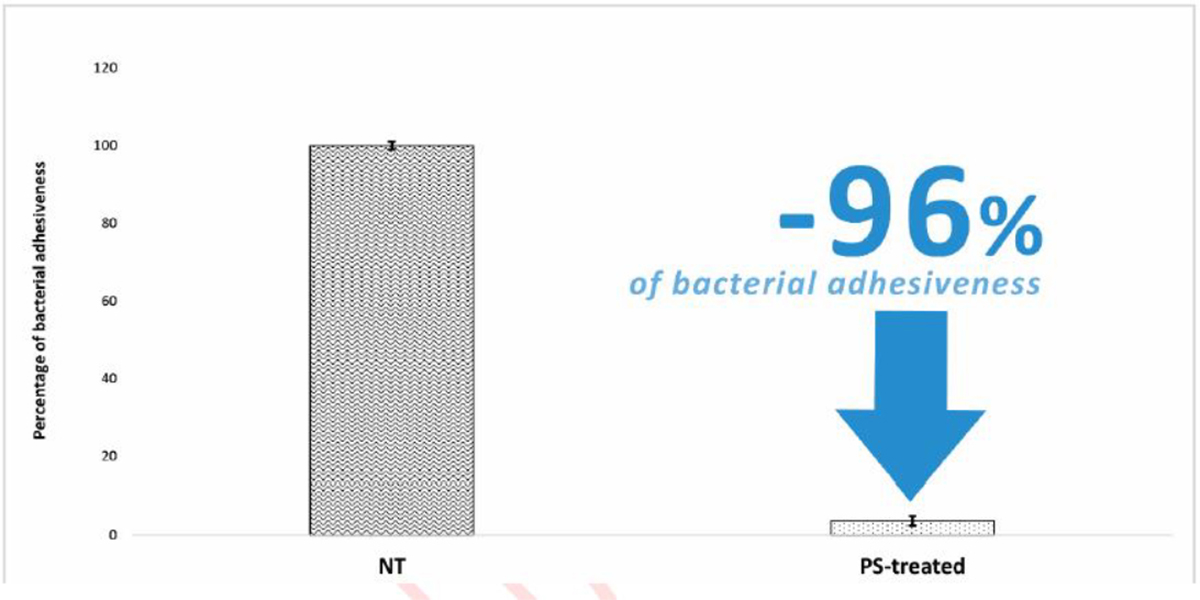
Percentage of *S. aureus* adhesiveness in PS-treated commercial pericardial T-GT bioprosthetic heart valve leaflets compared to untreated ones (NT). Data represent the means ± SD (n=6 for each group).

The PS-treatment significantly reduced the adhesiveness of *S. aureus* on the treated SBHVs (Figure 1). Results were expressed as the percentage decrease of the attached micro-organisms assessed in the PS-treated pericardial T-GT leaflets by comparison with the untreated ones. Specifically, PS treatment resulted in the inhibition of the adhesiveness of the *S. aureus* strain by 96%.

### 3.4 Assessment of the Non-Pyrogenicity of the PS-Treated Tissue

Table 3 reported the Endotoxin Unit evaluation in untreated and PS-treated commercial T-GT leaflets. The treatment with polyphenols does not support any contaminants capable of triggering a pyrogenic reaction. Reference Value to be considered pyrogenic: ≥ 20 EU/device.

## 4. Discussion

The epidemiological outline of IEs after surgical or transcatheter BHV implant is challenging and it quite differs by comparison to data from different international registries/studies, showing great variability and, sometimes, conflicting conclusions. The incidence rate of IEs (Table 4) seems to be slightly higher for TBHVs (from 0.52% to 3.25%) than for SBHVs (from 0.3% to 2.5%), however, what concerns most are the side effects and comorbidities linked to the onset of infections, often made even more life-threatening by the advanced age of the patient. Recently, Abegaz et al. reported a mortality rate for SBHVs-related IEs that ranged from 20% to 37% at short- and up to five-year follow-up, while the rate of complications due to septic embolisms, cardiac, and/or renal involvement ranged between 19% and 39% [14]. In addition, 25% of patients already treated for IE might be re-hospitalized, due to recurrent cardiac valve infection [15]. Luehr and colleagues [16] demonstrated overall in-hospital mortality equal to 22.3%, which increased to 25.2% during the follow-up period. Analyzing in detail the timing of progression of deaths related to the infection, it was reported that 14.8% occur after the first 30 days from the onset and 30.1% after 1 year [17]. This scenario is made even more dramatic by the occurrence of postoperative complications, such as permanent renal failure (20.4%), sepsis and/or systemic inflammatory response syndrome (27.2%), low cardiac output syndrome (15.5%), and the need for ECMO support (12.6%) [16]. The implantation of a TBHV is often performed on patients who already have a heart failure history [18], who suffer from paravalvular aortic regurgitation, that often needs implantable cardiac devices [19] maybe with previous sepsis or cardiac arrest episodes; moreover, the use of a non-hybrid surgical room [20] and a major risk of bleeding during hospitalization [18] contribute to the exposure to a greater risk of developing IEs. The FinnValve Registry reported a cumulative increase in mortality rate related to the onset of IEs after TBHV implant, ranging from 37.7% within 30-days after diagnosis to 52.5% one year after [21]. A systematic analysis from Khan and colleagues outlined in-hospital mortality that ranged from 11% to 47.2%, mortality rate at follow-up from 11% to 75%, and heart failure occurrence from 20% to 67.9% [22]. The PS reported an interesting microbicidal action even on particularly dangerous bacterial strains such as *M. chelonae*; noteworthy, that PS resulted in more effective than an ethanol-based solution and comparable to that of standard treatment with antibiotics (Table 1). The mechanisms of antimicrobial action of phenolic compounds are not yet been fully understood. Many papers explained this activity by the alteration of the cell membrane permeability, the changes in various intracellular functions influenced by hydrogen bonding of the phenolic compounds to enzymes, or by the modification of the cell wall rigidity with integrity losses due to different interactions with the cell membrane [23]. The effects are related to the induction of irreversible damage in the cytoplasmic membrane and coagulation of the cell content, inhibiting the functionality of the intracellular enzyme, energy metabolism, and DNA synthesis [24]. Furthermore, polyphenols may link to soluble proteins located on membranes forming complexes that prevent recognition with other membrane receptors [25]. The unexpected virucidal action of PS evaluated in this study (Table 2) could be explained by their ability to inhibit the recognition mechanisms of viral receptors responsible for docking on the cell membrane as well as damaging the protein envelope that contains the genetic material. Surprisingly, the polyphenols have proven to almost completely reduce the bacterial adhesiveness of *S. aureus* to PS-treated commercial BHVs leaflets making the technology even more clinically interesting and useful (Figure 1). Finally, regulatory agencies require that medical devices be tested for material-mediated pyrogenicity following “ISO 10993–11:2017 Biological evaluation of medical devices – Part 11: Tests for systemic toxicity”. The term pyrogen (Greek pyros: fire) defines fever-inducing substances. A pyrogenic response induced by a medical device may be due to several causes depending on the presence of so-called “material-mediated pyrogens”. One class of well-known and well-characterized exogenous pyrogens is the class of endotoxins, a lipopolysaccharide component present on the cell walls of Gram-negative bacteria. Another broad class of exogenous pyrogens is non-endotoxin pyrogens, which include substances such as lipoteichoic acid, originating from Gram-positive bacteria, and other compounds originating from fungi, yeast, viruses, bacteria, and parasites. The third class of non-endotoxin pyrogens is that material-mediated pyrogen. Although no formal definition of material-mediated pyrogens exists, it is thought that they may leach from medical device materials or surfaces. Material-mediated pyrogens may also stem from contamination introduced during manufacturing and packaging, such as residues from cutting fluids, mold releases, cleaning agents, and processing aids. Therefore, it is of fundamental importance to develop treatments, capable of counteracting the onset of IEs related to a BHV implant without introducing chemicals or contaminants responsible for rising pyrogenic reactions. The PS treatment has proven to be largely safe and can be classified as a non-pyrogenic treatment (Table 3).

## 5. Conclusions

The current *in-vitro* study highlights the protective action of polyphenols by preventing the adhesion of one of the bacterial strains most commonly associated with the onset of IEs. The very marked reduction of the ability of *S. aureus* to adhere to the treated commercial BHV leaflets was an interesting finding that warrants further investigation given the potential benefit of decreasing the incidence of BHV-related IE. Finally, the disinfectant-like property of the polyphenolic solution is also able to avoid any contamination during treatment, ensuring an environment suitable for the production of long-term implantable biomedical devices such as BHVs. The authors are investigating the impact of the treatment on the biomechanical characteristics of the treated tissue, preliminary data confirm that the polyphenol-based process maintains the appropriate elastic properties and mechanical resistance, currently, the data have been collected and are being processed. At this stage, PS can be identified as a promising technology capable of guaranteeing unprecedented improvements, based on the action of safe, well-known, and characterized conventional molecules as polyphenols, easy to apply and perfectly integrable into a BHV production line.

## Figures and Tables

**Table 1:** Percentage of microbicidal activity of PS on different strains of micro-organisms compared to standard antibiotics (50μg/ml of Gentamicin for bacterial strains and 1200μg/ml of Nystatin B for mycotic strain) and ethanol solution (10% v/v in phosphate buffer).

		% MICROBICIDAL ACTIVITY		
MICRO-ORGANISM		*PS*	*Antibiotics*	*Ethanol*
*Pseudomonas aeruginosa*	Gram −	99,999	99,999	99,99
*Salmonella enterica typhimurium*	Gram −	99,999	99,999	90
*Enterococcus faecalis*	Gram +	99,99	99,99	0
*Listeria monocytogenes*	Gram +	99,999	99,999	0
*Staphyloccoccus aureus*	Gram +	99,999	99,999	99
*Streptococcus viridans*	Gram +	99,999	99,999	90
*Candida albicans*	Yeast	99,999	99,999	99,9
*Aspergillus brasiliensis*	Fungi	99,9	99,999	90
*Mycobacterium chelonae*	Mycobacterium	99,999	99,999	99

**Table 2:** Percentage of virucidal activity of PS on different strains of viruses. Data represent the means ± SD.

Type of Virus	% Virus Inactivation
*Poliovirus Type 1 LSc-2ab – RVB1260*	99,01 ± 3.20
*Adenovirus Type 5 – ATCC VR5*	99,70 ± 1.02
*Murine norovirus S99 – RVB651*	99,48 ± 1.54

**Table 3:** Endotoxin Unit evaluation in untreated (NT, n=6) and PS-treated (PS, n=6) commercial T-GT leaflets. Reference Value to be considered pyrogenic: ≥ 20 EU/device.

Tissue Type	Endotoxin Unit/Sample
*NT*	< 0.666
*PS*	< 0.666

**Table 4:** Incidence rate of infective endocarditis related to Surgical (SBHVs) or Transcatheter (TBHVs) bioprosthetic heart valves as reported from different international registries or studies.

SBHVs related IEs			TBHVs related IEs		
Patients involved (n°)	Incidence	Reference	Patients involved (n°)	Incidence	Reference
4333	0.30%	20	7273	0.52%	33
60253	1.40%	16	1820	3.02%	34
8530	0.40%	33	4336	1.40%	35
66077	2.50%	19	33248*	3.25%	28

* Comprehensive meta-analysis.

## Abbreviations:

SBHV: Surgical Bioprosthetic Heart Valve
TBHV: Transcatheter Bioprosthetic Heart Valve
PS: Polyphenolic Solution
CFU: Colony-Forming Units
ECMO: Extracorporeal Membrane Oxygenation
